# Unveiling primary Hyperoxaluria type 1: a fortuitous discovery through bone marrow biopsy

**DOI:** 10.1093/omcr/omae128

**Published:** 2024-10-26

**Authors:** Taha Yassine Aaboudech, Kaoutar Znati, Ahmed Jahid, Samia Sassi, Salima Driouich, Fouad Zouaidia, Zakia Bernoussi

**Affiliations:** Pathology Department, Ibn Sina Hospital, Rabat, Morocco; Mohammed V University in Rabat, Rabat, Morocco; Pathology Department, Ibn Sina Hospital, Rabat, Morocco; Mohammed V University in Rabat, Rabat, Morocco; Pathology Department, Ibn Sina Hospital, Rabat, Morocco; Mohammed V University in Rabat, Rabat, Morocco; Pathology Department, Ibn Sina Hospital, Rabat, Morocco; Mohammed V University in Rabat, Rabat, Morocco; Pathology Department, Ibn Sina Hospital, Rabat, Morocco; Department of Internal Medicine Hematology and Geriatrics, Ibn Sina Hospital, Rabat, Morocco; Pathology Department, Ibn Sina Hospital, Rabat, Morocco; Mohammed V University in Rabat, Rabat, Morocco; Pathology Department, Ibn Sina Hospital, Rabat, Morocco; Mohammed V University in Rabat, Rabat, Morocco

**Keywords:** bone marrow biopsy, haematology, histopathology, oxalate crystals, primary hyperoxaluria

## Abstract

This paper details a rare case of primary hyperoxaluria type 1 (PH1) identified through a bone marrow biopsy in a 46-year-old female patient with a history of nephrolithiasis and chronic renal failure. Genetic analysis identified the p.Ile244Thr mutation in the AGXT gene, confirming the diagnosis of PH1. The paper aims to highlight this case, focusing on the genetic basis of the disorder, including the identified mutation. It underscores the importance of early diagnosis of infantile and childhood nephrolithiasis, particularly in cases with familial history, to prevent renal loss and systemic oxalosis.

## Introduction

Primary hyperoxaluria (PH) is a rare genetic disorder resulting in excessive oxalate production due to a metabolic defect in the glyoxylate pathway, leading to elevated serum oxalate levels and subsequent oxaluria. This condition is characterized by the widespread deposition of calcium oxalate crystals (oxalosis) in various organs. Oxalosis occurs in both renal and extrarenal tissues [1]. Primarily, calcium oxalate accumulates in the renal tubulointerstitium, causing acute and chronic tubulointerstitial nephritis, nephrolithiasis, and eventually renal failure. The crystals can then migrate to the bone marrow and other tissues, replacing marrow parenchyma and causing pancytopenia and a leukoerythroblastic reaction [2]. PH exists in three genetic forms (types 1, 2, and 3), with Type 1 being the most severe due to mutations in the AGXT gene, involving liver enzymes and an aggressive disease pattern [3]. This report describes a case of primary hyperoxaluria type 1, initially diagnosed via bone marrow biopsy in a 45-year-old woman with anemia and end-stage renal disease (ESRD). It is notable that primary hyperoxaluria is typically not diagnosed through bone marrow biopsy.

## Case report

A 45-year-old woman, with no known consanguinity, has a family history of kidney stones. Her mother died from an unspecified nephropathy while undergoing hemodialysis. Since the age of 8, she has experienced recurring kidney stones, leading to the progression of chronic renal failure. Over the past 4 years, she has undergone thrice-weekly hemodialysis due to nephrocalcinosis and has been placed on the waitlist for a kidney transplant. She also suffers from chronic polyarthritis with ecchymosis on the lower limbs and conjunctival hemorrhages.

Recently, she was referred to the hematology department due to debilitating bone pain, persistent fatigue, and a gradual onset of anemia persisting for 1 year. Notably, there was no response to erythropoietin, and hepatosplenomegaly was absent. Laboratory tests showed normochromic, normocytic anemia with a hemoglobin level of 10.2 g/dl, a leukocyte count of 7100/mm^3^, and a platelet count of 129 000/mm^3^. The reticulocyte count was 3.2%. Serum biochemistry revealed elevated ferritin levels, along with increased blood urea (105 mg/dl) and serum creatinine (1.65 mg/dl). Liver enzymes were within normal limits, and viral markers were negative. The phosphocalcic balance was normal, but the level of alkaline phosphatase was elevated (694 UI/l).

**Figure 1:**
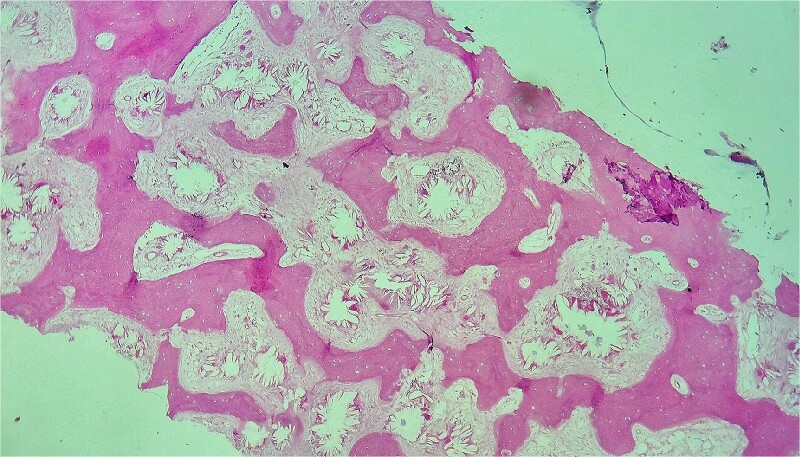
Bone marrow biopsy demonstrating extensive calcium oxalate crystals arranged in stars or rosettes. Hematoxylin & Eosin stain, × 10.

**Figure 2:**
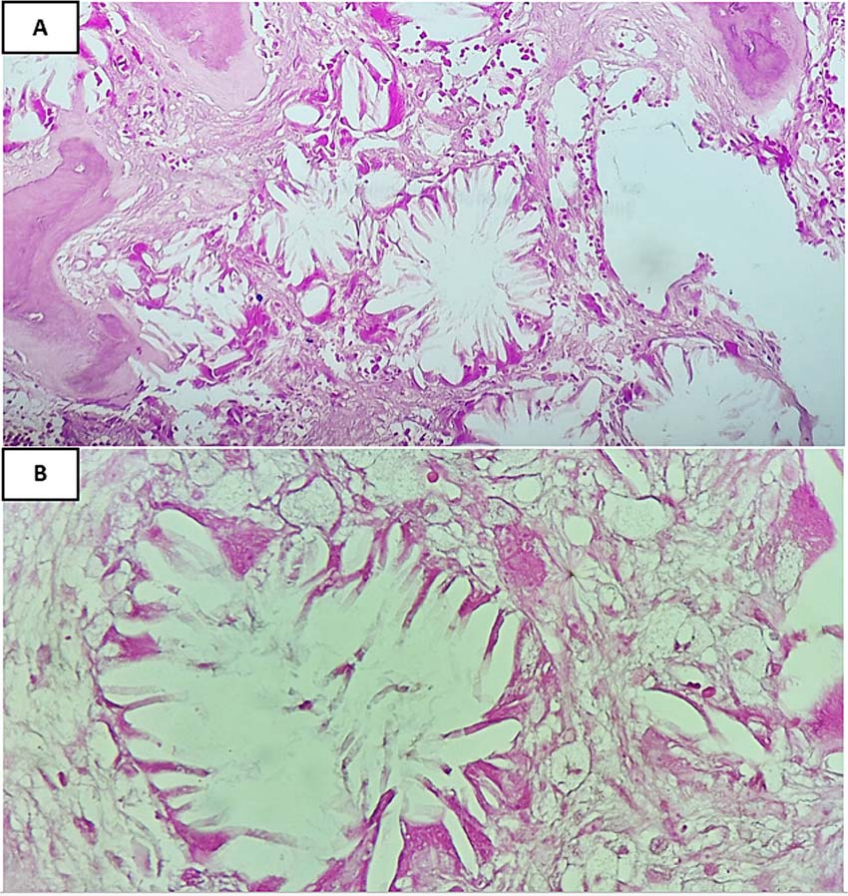
Calcium oxalate crystals are surrounded by a vigorous foreign body giant cell reaction. (A) Hematoxylin & Eosin stain, ×20) and (B) Hematoxylin & Eosin stain, ×40).

**Figure 3:**
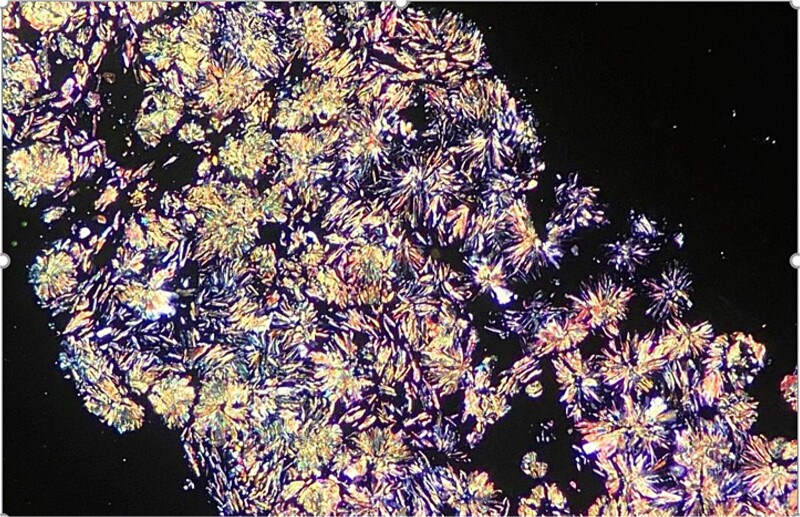
Polarized light examination showed a distinctive birefringence of the oxalate crystals.

A computed tomography scan confirmed nephrocalcinosis without hepatosplenomegaly and showed generalized hyperdensity of the bones. Bone marrow aspirate resulted in a dry tap. The bone marrow biopsy, measuring 1.5 cm, displayed six bone chambers extensively invaded by grayish-colored oxalate crystals arranged in stars or rosettes. This crystal deposition was surrounded by a vigorous foreign body giant cell reaction, with fibrotic areas exhibiting fibroblastic proliferation and irregular trabecular bone remodeling (Fig. 1, Fig. 2A, and Fig. 2B). Polarized light examination demonstrated a marked pale green birefringence of the oxalate crystals (Fig. 3). Subsequent plasma oxalate was correct but urinary oxalate levels were elevated (62 mg/24 h). Additionally, a cardiac ultrasound, combined with magnetic resonance imaging (MRI), identified myocardial deposits suspected to be either oxalate deposition or thrombosis.

Mutation analysis identified homozygosity for the p.Ile244Thr mutation in exon 7 of the AGXT gene. Based on these findings, a diagnosis of primary hyperoxaluria type I was established.

## Discussion

PH1 is a rare autosomal-recessive condition with an estimated prevalence of 1 to 3 cases per million and an incidence rate of around 1.1 per 100 000 live births annually in Europe [4]. Its frequency is higher in areas where consanguineous marriages are common. Reports suggest that approximately 10% of Kuwaiti and 13% of Tunisian children with end-stage renal disease are diagnosed with primary hyperoxaluria. This disorder is caused by a deficiency of alanine-glyoxylate aminotransferase in the liver’s peroxisomes, leading to excessive oxalate production [5].

PH is categorized by three enzyme deficiencies that impair oxalate metabolism. The most common form, PH type 1 (PH1), accounting for 80% of cases, results from a deficiency in the Vitamin B6-dependent hepatic peroxisomal enzyme Alanine Glyoxalate Aminotransferase (AGT). This enzyme converts L-alanine and glyoxalate into pyruvate and glycine. The enzyme defect is due to mutations in the AGXT gene on chromosome 2, leading to hyperoxaluria. PH2 and PH3 are caused by deficiencies in glyoxylate reductase/hydroxypyruvate reductase (GRHPR) and the mitochondrial enzyme HOGA1, respectively [3].

The oversaturation of calcium oxalate results in recurrent urolithiasis and/or nephrocalcinosis. Renal impairment decreases oxalate elimination, causing its deposition in various tissues, a condition termed systemic oxalosis [6].

Bone marrow (BM) oxalosis is a rare finding. Few cases in the literature describe it as a form of systemic oxalosis involving oxalate deposition in the BM, which can lead to cytopenias, leukoerythroblastosis, and hepatosplenomegaly. BM findings typically include birefringent calcium oxalate crystals visible under polarized microscopy and granulomatous structures. [7]. Typically, a bone marrow biopsy is not used to diagnose hyperoxaluria but is employed to investigate treatment-resistant anemia or to diagnose renal osteodystrophy in dialysis patients [2].

More than 175 mutations in the AGXT gene have been identified, with several common variants in PH1. Key mutations include c.33dupC, p.Gly170Arg (c.508G- > A), and p.Ile244Thr (c.731 T- > C), which are found in at least one of the two affected alleles in approximately 70% of PH1 cases. Most AGXT mutations lead to reduced or eliminated AGT activity, and some mutations redirect the enzyme to mitochondria instead of peroxisomes. Though the mitochondrial enzyme remains active, it cannot access peroxisomal glyoxylate [8]. Generally, there is limited correlation between AGXT mutations and ethnicity, except for the Ile244Thr mutation. Known as the Maghrebian mutation, it originates from North Africa and is the most prevalent mutation, representing up to 91.6% of AGXT mutations in North African countries [9]. This aligns with our genetic analysis findings in this case.

Early detection and appropriate conservative therapy are crucial to prevent the severe outcomes associated with cytopenias if full-blown marrow failure occurs. The recommended treatment for systemic oxalosis includes kidney transplantation or a combined liver and kidney transplant [10].

## Conclusion

Bone Marrow oxalosis is a rare finding. Physicians should include it as a potential alternative diagnosis when evaluating patients with renal failure and metabolic irregularities, particularly in childhood cases. Additionally, in regions such as North Africa where consanguineous marriages are common and the Ile244Thr mutation in the AGXT gene is prevalent, parents of those with primary oxalosis should be offered genetic counseling. Timely identification and appropriate conservative treatment are crucial to prevent anticipated complications arising from cytopenias.

## Consent

A written consent for publication has been obtained from the patient.

## Guarantor

Taha Yassine Aaboudech is the guarantor of this article.
